# Genetic screening in sporadic ALS and FTD

**DOI:** 10.1136/jnnp-2017-315995

**Published:** 2017-06-22

**Authors:** Martin R Turner, Ammar Al-Chalabi, Adriano Chio, Orla Hardiman, Matthew C Kiernan, Jonathan D Rohrer, James Rowe, William Seeley, Kevin Talbot

**Affiliations:** 1 Nuffield Department of Clinical Neurosciences, Oxford University, Oxford, UK; 2 Department of Basic and Clinical Neuroscience, King’s College London, London, UK; 3 Department of Neuroscience, University of Torino, Torino, Italy; 4 Academic Unit of Neurology, Trinity College Dublin, Dublin, Ireland; 5 Brain and Mind Centre, Royal Prince Alfred Hospital, Sydney, New South Wales, Australia; 6 Dementia Research Centre, UCL Institute of Neurology, London, UK; 7 Department of Clinical Neurosciences, University of Cambridge, Cambridge, UK; 8 Department of Neurology, Memory and Aging Center University of California San Francisco, San Francisco, California, USA

**Keywords:** ALS, C9ORF, FRONTOTEMPORAL DEMENTIA, GENETICS

## Abstract

The increasing complexity of the genetic landscape in amyotrophic lateral sclerosis (ALS) and frontotemporal dementia (FTD) presents a significant resource and physician training challenge. At least 10% of those diagnosed with ALS or FTD are known to carry an autosomal dominant genetic mutation. There is no consensus on what constitutes a positive family history, and ascertainment is unreliable for many reasons. However, symptomatic individuals often wish to understand as much as possible about the cause of their disease, and to share this knowledge with their family. While the right of an individual not to know is a key aspect of patient autonomy, and despite the absence of definitive therapy, many newly diagnosed individuals are likely to elect for genetic testing if offered. It is incumbent on the practitioner to ensure that they are adequately informed, counselled and supported in this decision.

## Introduction

High on the list of questions for those diagnosed with amyotrophic lateral sclerosis (ALS), frontotemporal dementia (FTD) and other neurodegenerative disorders is whether the disease is hereditary. It may be easy for the clinician to overlook the strength of an individual’s desire to understand the factors leading to their disease, including genetic causes, even if this does not change specific treatment. If no relevant family history is revealed by a newly diagnosed individual with ALS where good knowledge of the family exists, one study has suggested that their children have almost the same chance of not developing ALS as the general population.^1^ In the context of conveying the news of a terminal diagnosis, the desire to offer some aspect of reassurance will be strong. However, this does not obviate the need to consider the role of genetic testing.

A previous review concluded, based on knowledge current at that time, that testing should not be offered to those with sporadic ALS.^2^ An increased appreciation of the major limitations of family history taken in the clinic, rapid advances in preimplantation screening, increasing availability of commercial genetic testing and the promise of gene-targeted therapy make it timely to reconsider this position. This article considers the arguments for and against offering routine genetic testing to all those with sporadic ALS or FTD.

### The genetic landscape of ALS and FTD

**Figure 1:**
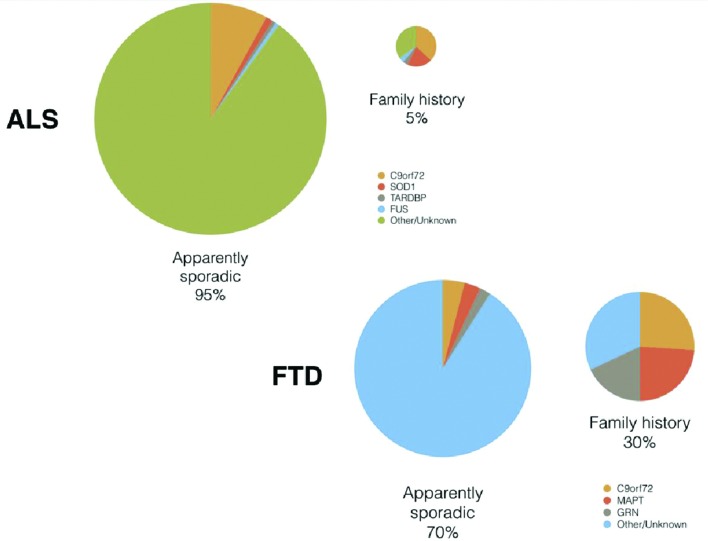
Upper panel: Comparison of proportions of monogenic causes of ALS in those reporting a family history of ALS versus apparently sporadic cases. Lower panel: Comparison of proportions of monogenic causes of FTD in those reporting a family history of dementia versus apparently sporadic cases. ALS, amyotrophic lateral sclerosis; FTD, frontotemporal dementia.

Clinical, pathological and genetic overlap between ALS and FTD is now well established.^3^ Neuronal and glial cytoplasmic inclusions containing the 43 kDa transactive response DNA binding protein, TDP-43, are found in 98% of all cases of ALS and approximately 50% of FTD.^4^ A hexanucleotide expansion (G4C2) in intron 1 of the *C9orf72* gene is the cause of chromosome 9-associated^5 6^ and linked^7 8^ pure ALS and FTD (typically the behavioural variant), and mixed ALS-FTD,^9 10^ with multiple phenotypes seen within the same pedigree.^11^ Based on meta-analysis of international cohorts (mainly Western hemisphere), 5% of those with ALS are recorded as familial.^12^ In FTD, this number is higher, at approximately 25%–30%. Approximately one-third of ALS and FTD cases will harbour a pathogenic *C9orf72* expansion. Screening of individuals with ALS or FTD, but without an apparent family history of either, reveals up to 10% as carriers of the expansion. While there are about 30 genes in which variation has been repeatedly associated with ALS, these account collectively for a much smaller proportion of cases.^13^ Fewer genes have been associated with FTD, but mutations in *GRN* and *MAPT* are common causes, although less frequent than *C9orf72* expansions. Although both ALS and FTD are typically disorders of middle-to-late life, some genes, for example *FUS*, have been associated with juvenile forms^14^ (see figure 1).

### The clinical distinction between familial and sporadic disease is unreliable

The distinction between familial and sporadic disease can be influenced by ascertainment bias, and there is a growing appreciation of the limitations of family history taking in the clinic, compounded by a lack of a uniform definition of familial disease.^15 16^ Boundaries will also widen significantly with the inclusion of related disorders (including autism, schizophrenia, bipolar disease, multiple sclerosis and Parkinson’s disease^17^). A reliable family history of cognitive disorders is another challenging area, specifically differentiating Alzheimer’s disease from FTD in relatives based on clinical history alone, with a risk of inflating or underestimating the frequency of a positive family history.

A single gene variation may result in different phenotypes, which may reduce ascertainment if the alternative phenotypes are not recognised as relevant. *C9orf72* expansions have been associated with diagnoses of dementia (including behavioural variant FTD, primary progressive aphasia and even Alzheimer-type dementia), ataxia, chorea and schizophrenia. Such disorders may well be overlooked by respondents when asked about their family history in the context of an ALS assessment. The penetrance of many ALS genes is age-dependent and incomplete, and family size directly influences the probability of an affected relative.^18^

The distinction between familial and sporadic ALS is therefore not clear-cut, and this is underscored by the observation that every established familial ALS gene has also been implicated in sporadic ALS.^19^ Penetrance, the probability of manifesting a phenotype given one is a carrier of the risk genotype, is closely related to the frequency and effect size of disease-associated gene variants. Genotypes associated with high penetrance typically have very large effect sizes and are usually rare. Low penetrance genotypes are usually associated with a small effect. For several ALS genes, including the *C9orf72* expansion mutation, penetrance has been reported as high within affected families,^11 20^ but this is not what is expected from observation of the ratio of familial and sporadic frequencies in the UK,^21^ which predicts an overall penetrance of 38% (http://alsod.iop.kcl.ac.uk/misc/penetrance.aspx), nor from the very strong genetic association signal in large studies of people with apparently sporadic ALS.^22^ Incomplete penetrance, and the current lack of a gold standard for how to measure it, complicates decisions of whether to test in the absence of a family history and how to interpret a positive result. A further complexity is the rare occurrence of de novo mutations, which has been described in *FUS* 14 and *SOD1*.^23^

### The argument against routinely offering *C9orf72* testing

At present, there is no disease-modifying or neuroprotective therapy for *C9orf72*-related disease, and uncertainty remains over factors influencing penetrance, which may vary between individual families. Consent to test cannot be truly informed if there is insufficient information. Although clinical trials are underway, until an effective therapy is available, a positive test for the *C9orf72* expansion in someone with no family history of ALS or FTD has life-changing implications for relatives. There is therefore understandable concern that cannot yet be offset by the prospect of an effective treatment. There will be consequent pressure for consideration of presymptomatic testing, the counselling for which requires high-quality evidence. Individuals might be strongly advised to involve other family members in their decision to undergo testing, but there is a risk that a healthy individual’s right *not* to know about their own risk might be inadvertently breached. Uncertainty is compounded by the difficulty in interpretation of a positive gene test in the absence of a relevant family history, since there will have been many obligate carriers who did not manifest disease or who developed a different but related condition.

### The argument for offering *C9orf72* testing routinely

It is now possible for at least 10% of all those diagnosed with ALS and FTD to understand the cause of their disease, and to share this knowledge with relatives to allow them to make decisions about learning their own and any future children’s genetic status. For the symptomatic individual, it can bring a degree of understanding and accommodation to what is otherwise a random and unexplained blow.

For the newly diagnosed individual, a clinician may be tempted to withhold information or defer testing because knowledge is incomplete, or through concern that the individual or their family will be further burdened in the absence of a therapy. In many countries, there are no longer healthcare system barriers to accessing personal genetic testing, and the provision of impartial and evidence-based information by experienced clinicians is preferable to individuals seeking information in an unguided, unfiltered way through the internet.

Newly diagnosed individuals may not wish to burden their children with worries over the future, but might equally be keen to offer them the option to consider preimplantation screening, especially in circumstances where it could be undertaken without disclosing the presence of an obligate carrier.

Finally, established international research consortia such as the Presymptomatic Neurodegeneration Initiative and Genetic Frontotemporal Dementia Initiative anticipate exciting new therapeutic options. For example, antisense oligonucleotide therapy against wild-type *SOD1* is about to enter its first trials in ALS, and similar therapies for *C9orf72* expansions are likely in the next few years. Information about anticipated developments that are directly relevant to known mutations may be a compelling part of the decision-making regarding genetic testing for some newly diagnosed individuals.

The imminent availability of therapy has also been identified as a major factor in changing physician practice in relation to routine testing,^23^ although benefit from genetic therapy in established neurodegenerative disorders remains unproven, and the demonstration of efficacy in disease prevention is likely to be years away.

It is noteworthy that a survey of 167 clinicians from 21 different countries (the majority of whom identified themselves as having a specialist interest in ALS) revealed that more than half would seek genetic testing if they had personally received the diagnosis.^24^ However, it must also be noted that there are practical issues in relation to the clinical expertise needed to provide nuanced and tailored conversations with individuals from diverse backgrounds and to ensuring that resources are equally available to all and understandable across a range of educations and backgrounds. There are also issues in relation to the financial cost to the individual of any testing. Importantly, many neurologists lack the specialised training of clinical geneticists. Familiarity with the complex issues involved and knowledge of who to refer to specialist services is therefore vital for specialists and trainees^25^ to fully realise the benefits of the rapid genetic advances for all those diagnosed with ALS and FTD, and their families.

## Conclusions

Across the clinical neurosciences, there is increased understanding of the need to provide expert interpretation of freely available scientific knowledge. It is incumbent on the medical profession to recognise patient autonomy and to support decision-making by those who strongly believe that they are maximising options for their own children and wider family, as well as trying to understand their own disease, while also recognising the right not to know one’s genetic status.^26^ Either way, the practice of refraining from any discussion of genetic testing should now be challenged as an unnecessary limitation to the provision of best care in ALS and FTD.
